# Systemic Lymphadenopathic Mastocytosis with Eosinophilia

**DOI:** 10.3390/diagnostics12123057

**Published:** 2022-12-06

**Authors:** Soyoung Im, Jeong-A Kim, Gyeongsin Park, Uiju Cho

**Affiliations:** 1 Department of Pathology, St. Vincent’s Hospital, College of Medicine, The Catholic University of Korea, Seoul 06591, Republic of Korea; 2 Department of Internal Medicine, St. Vincent’s Hospital, College of Medicine, The Catholic University of Korea, Seoul 06591, Republic of Korea; 3 Department of Pathology, Seoul St. Mary’s Hospital, College of Medicine, The Catholic University of Korea, Seoul 06591, Republic of Korea

**Keywords:** mastocytosis, systemic mastocytosis, lymphadenopathy, eosinophilia

## Abstract

Systemic mastocytosis is a neoplastic proliferation of mast cells that most frequently involves cutaneous sites. Mastocytosis involves various extracutaneous sites, but the lymph node is rare. We present an interesting image of systemic mastocytosis in the lymph node with marked eosinophilia. It is a rare subtype of systemic mastocytosis requiring high suspicion levels for the correct diagnosis.

**Figure 1:**
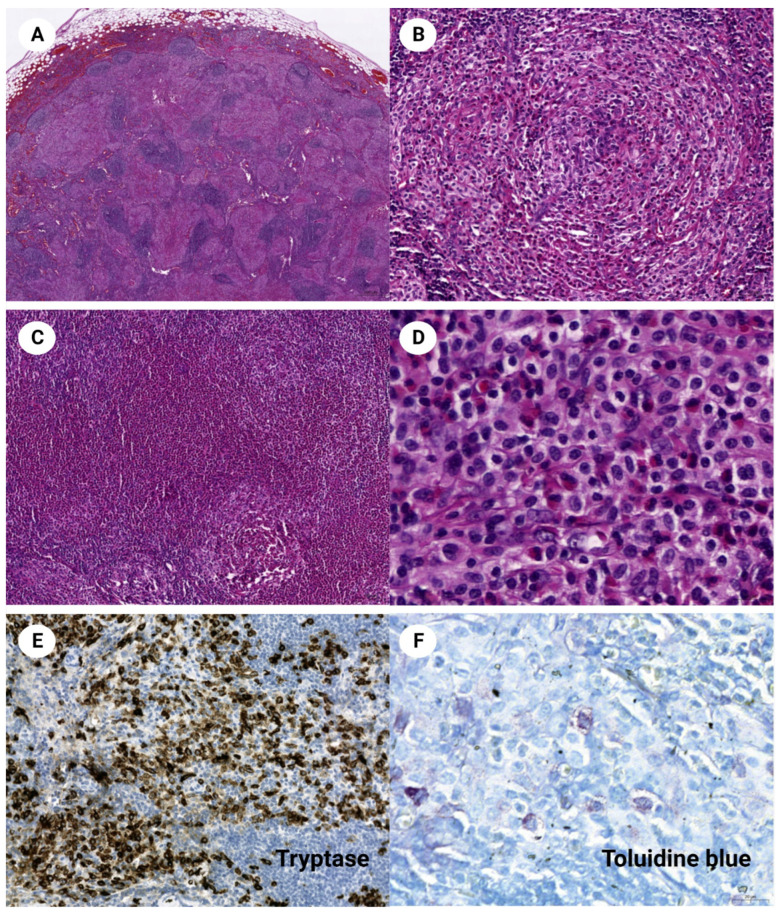
The lymph node demonstrates an interfollicular and sinusoidal proliferation of pale oval cells and abundant eosinophilic aggregates (**A**, H&E stain, ×20). It has a granuloma-like pattern with delicate sclerosis (**B**, H&E stain, ×200) and eosinophilic microabscesses (**C**, H&E stain, ×100). The neoplastic cells have medium-sized with clear or pinking moderate cytoplasm. The nucleus of some cells shows a vesicular nuclear membrane and distinguishable nucleoli (**D**, H&E stain, ×630). The neoplastic cells are confirmed as mast cells expressing tryptase (**E**, ×200) and having basophilic cytoplasmic granules (**F**, toluidine blue stain, ×630).

**Figure 2:**
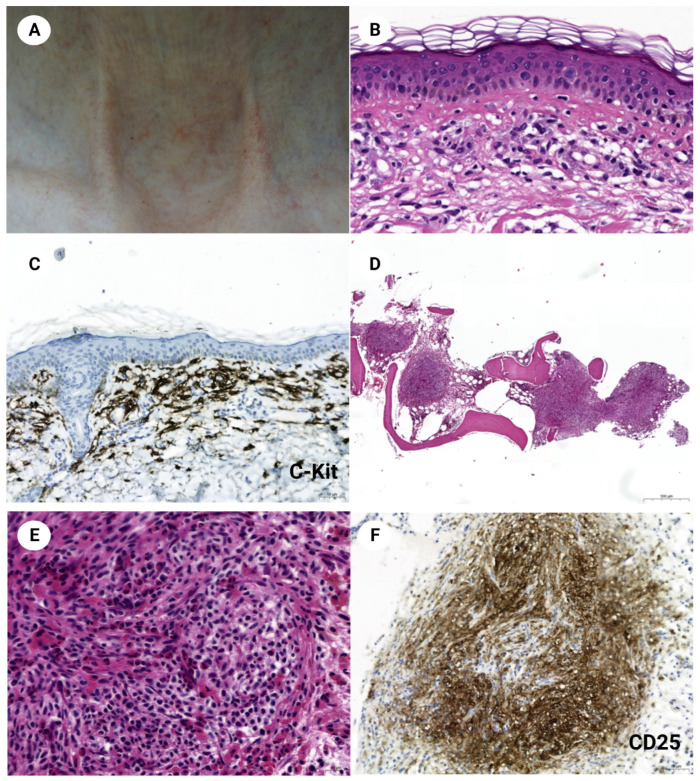
The patient has multiple purpuric telangiectases around the neck skin (**A**). Mast cells with basophilic cytoplasmic granules are increased in the upper dermis of the lesion (**B**, H&E stain, ×400). C-Kit immunohistochemical stain highlights the infiltrating mast cells (**C**, ×200). In the bone marrow, multifocal compact aggregates of masts cells with paratrabecular and interstitial distributions are seen (**D**, H&E stain ×35). The abnormal mast cells contain spindle-shaped nuclei and clear cytoplasm and are associated with thin sclerotic fibers (**E**, H&E stain, ×400). CD25, which is not expressed in normal mast cells, is positive in these abnormal mast cells (**F**, ×200). A 64-year-old female patient was referred to the internal medicine clinic with an incidentally found mild splenomegaly and lymphadenopathy in the abdominal cavity during a health examination. She complained of mild abdominal discomfort and had a history of tuberculosis and cholecystectomy. Laboratory testing revealed a white blood cell count of 5.05 × 10^9^/L with 26.3% eosinophils. Segmented neutrophils and lymphocytes were 39.4% and 30.1%, respectively. Hemoglobin was 11.1 g/dL, platelet count was 186 × 10^12^/L, and LDH was 90 U/L. Abdominal CT demonstrated lymphadenopathy along the entire abdominal cavity, especially in the mesenteric and paraaortic areas. They measured less than 0.7 cm in short diameter. Mesenteric lymph node excision was performed for the diagnosis. The lymph node revealed aggregates and infiltration of pale-staining cells, largely replacing interfollicular and sinusoidal spaces. Nodular aggregates of these cells resembled granuloma. They were accompanied by delicate sclerosis and numerous eosinophils (Figure 1A–D). Tumor cells expressed C-kit and tryptase (Figure 1E), but were negative for CD1a, langerin, S100, CD21, CD3, CD20, CD34, CD30, and myeloperoxidase. Toluidine blue stain showed scanty basophilic cytoplasmic granules (Figure 1F), but the granules were not apparent in the Giemsa stain. Accordingly, the diagnosis of systemic mastocytosis was favored. The subsequent physical examination discovered multiple purpuric telangiectases around the neck and chest skin (Figure 2A). The skin biopsy demonstrated mast cells with small basophilic granules infiltrating the upper dermis (Figure 2B). The bone marrow biopsy contained a nodular aggregate of atypical mast cells that were positive for CD25 and CD117, confirming the neoplastic nature of the mast cells (Figure 2D–F). Like the lymph node, abundant eosinophilic infiltration was prominent in the bone marrow. Cytogenetic analysis of the bone marrow revealed a normal female karyotype, and real-time PCR molecular studies showed a lack of *BCR-ABL1* fusion, *PDGFRa*, and *PDGFRb* mutation. *KIT* gene harbored D816V mutation. Next-generation sequencing of the lymph node also demonstrated c.2447A>T p.(D816V) (VAF = 13.8%) mutation. The patient fulfilled the diagnostic criteria of systemic mastocytosis. It could be clinically subclassified as smoldering systemic mastocytosis because the patient had no C (‘cytoreduction-requiring’) findings and no evidence of an associated hematological neoplasm [1,2]. The patient is under watchful follow-up without active treatment and is stable one year after the diagnosis. Systemic mastocytosis is usually discovered initially in the bone marrow, and histology of the lymph node lesion is less familiar [3,4]. Lymphadenopathic mastocytosis with eosinophilia is a rare group of systemic mastocytosis that presents in ~10% of patients with systemic mastocytosis [5,6]. Clinical and morphologic features may be similar to myeloid and lymphoid neoplasms with PDGFRA rearrangement, and differential diagnosis between the two diseases by molecular studies is essential [7]. In the earlier classification of mastocytosis, lymphadenopathic systemic mastocytosis with eosinophilia was regarded as a separate mastocytosis category and categorized under aggressive systemic mastocytosis [8]. However, this term is no longer an official diagnosis in the current classification system [1,2]. Blood eosinophilia was shown to have a clinical significance, i.e., correlation with lymphadenopathy, dysmyeolopoiesis, WHO classification, and poor overall survival [9,10]. The present case demonstrates the classic yet rare image of lymphadenopathic mastocytosis. Granuloma-pattern morphology and abundant eosinophils could lead to other diagnoses, such as Langerhans cell histiocytosis. However, the correct diagnosis could be easily reached if clinicians and pathologists are aware of the diverse presentations of systemic mastocytosis.

## Data Availability

Data is contained within the article.
